# *Tyrannobdella rex* N. Gen. N. Sp. and the Evolutionary Origins of Mucosal Leech Infestations

**DOI:** 10.1371/journal.pone.0010057

**Published:** 2010-04-14

**Authors:** Anna J. Phillips, Renzo Arauco-Brown, Alejandro Oceguera-Figueroa, Gloria P. Gomez, María Beltrán, Yi-Te Lai, Mark E. Siddall

**Affiliations:** 1 Department of Biology, The Graduate Center, The City University of New York, New York, New York, United States of America; 2 Sackler Institute of Comparative Genomics, American Museum of Natural History, New York, New York, United States of America; 3 School of Medicine, Universidad Peruana Cayetano Heredia, Lima, Perú; 4 Department of Microbiology, Universidad Peruana Cayetano Heredia, Lima, Perú; 5 Enteroparasitology Laboratory, Peruvian Public Health Center, Peruvian Health Institute, Lima, Perú; 6 Institute of Zoology, National Taiwan University, Taipei, Taiwan; American Museum of Natural History, United States of America

## Abstract

**Background:**

Leeches have gained a fearsome reputation by feeding externally on blood, often from human hosts. Orificial hirudiniasis is a condition in which a leech enters a body orifice, most often the nasopharyngeal region, but there are many cases of leeches infesting the eyes, urethra, vagina, or rectum. Several leech species particularly in Africa and Asia are well-known for their propensity to afflict humans. Because there has not previously been any data suggesting a close relationship for such geographically disparate species, this unnerving tendency to be invasive has been regarded only as a loathsome oddity and not a unifying character for a group of related organisms.

**Principal Findings:**

A new genus and species of leech from Perú was found feeding from the nasopharynx of humans. Unlike any other leech previously described, this new taxon has but a single jaw with very large teeth. Phylogenetic analyses of nuclear and mitochondrial genes using parsimony and Bayesian inference demonstrate that the new species belongs among a larger, global clade of leeches, all of which feed from the mucosal surfaces of mammals.

**Conclusions:**

This new species, found feeding from the upper respiratory tract of humans in Perú, clarifies an expansion of the family Praobdellidae to include the new species *Tyrannobdella rex* n. gen. n.sp., along with others in the genera *Dinobdella, Myxobdella, Praobdella* and *Pintobdella*. Moreover, the results clarify a single evolutionary origin of a group of leeches that specializes on mucous membranes, thus, posing a distinct threat to human health.

## Introduction

**Figure 1:**
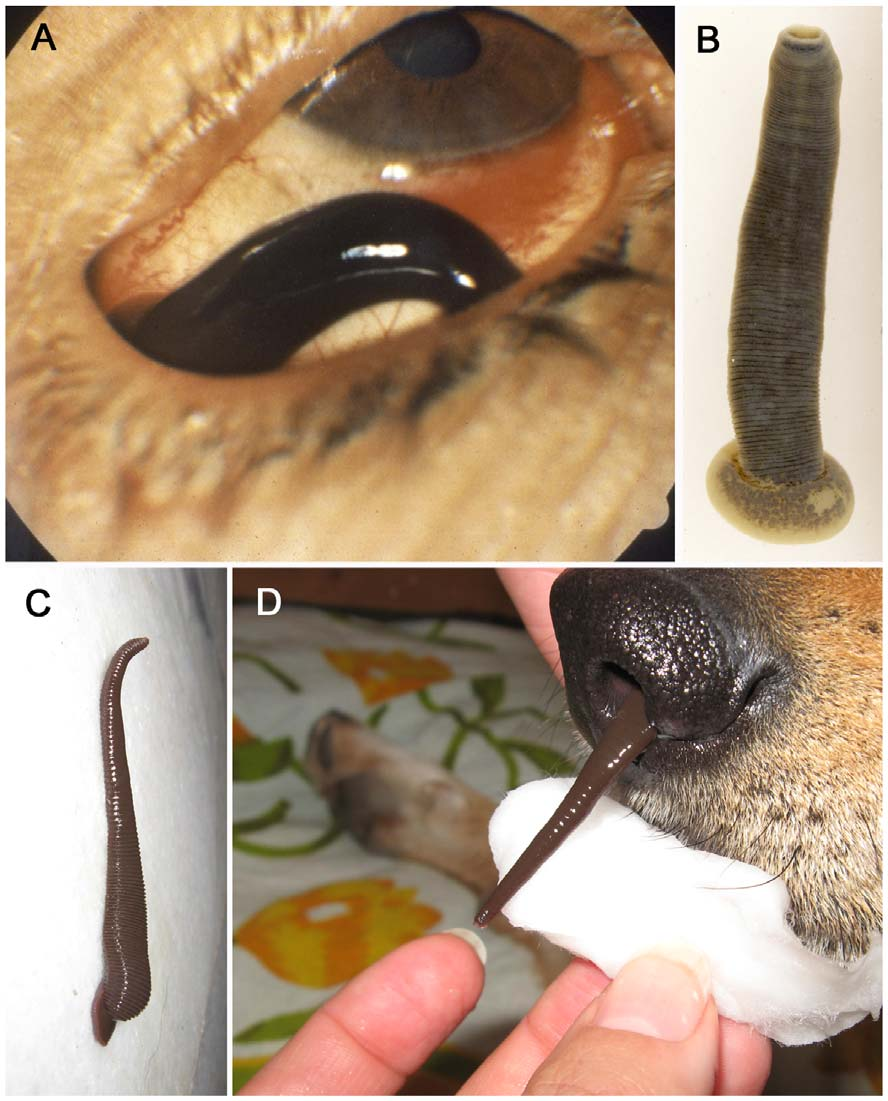
Mucosally invasive hirudinoid leeches. Known from a wide variety of anatomical sites including eyes (A) as in this case involving *Dinobdella ferox* (B), mucosal leech species, as in a case involving *Myxobdella annandalei* (C), more frequently feed from the nasopharyngeal surfaces of mammals (D).

Most people realize that they are being parasitized by a leech upon finding the worm attached to their skin. Disturbingly, leeches occasionally enter human orifices–a condition known as mucosal, orifical, vesical, or internal hirudiniasis depending on the localization of the leech (Fig. 1). Whereas most bloodfeeding leeches feed as ecto-parasites for short periods of time, those that feed on mucous membranes have been known to stay in an orifice for days or weeks on end [1], [2]. Cases of hirudiniasis are underreported because patients suffering from orificial hirudiniasis may only resort to medical attention if they are personally unsuccessful in extracting the leech [3]. Whereas invasive leeches are usually found in the nasopharyngeal region, there are many cases of leeches infesting various body orifices such as the eyes, urethra, vagina, or rectum [4]. Depending on the exact site of the bite in the nasopharyngeal region, symptoms may include hemorrhaging, hemoptysis, dysphonia, coughing, a tickling sensation, dyspnea or, in extreme cases, severe anemia and death [5], [6]. Hemorrhaging from leeches in the urethra, or even in the bladder, also poses a particular problem in that clot formation is inhibited by urine flow [7]. Underlying conditions, such as coagulation disorders or secondary bacterial infections can cause a patient's condition to escalate from relatively minor to life-threatening very quickly [2], [8], [7].

Reported cases of human orificial hirudiniasis are most common in rural areas of the Middle East, Africa, and Asia, however cases have been recorded from almost all continents [2]. Domestic and wild mammals in these regions, especially livestock, are at the greatest risk for orificial hirudiniasis in relation to the amount of time such animals spend at leech-inhabited watering holes [1]. Some species are more likely to afflict humans, such as *Dinobdella ferox* (Blanchard, 1896; literally translated to “terrible ferocious leech”), or species of *Limnatis, Praobdella*, and *Myxobdella* [9].

Leech systematics has been transformed with the addition of comparative sequence data and most of the groups historically recognized by taxonomists have been redefined or eliminated [e.g., 10, 11]. That said, Phillips and Siddall expressed reluctance to fully reorganize the systematics of New World hirudinoid leeches under Semiscolescidae in light of the unexpected finding of several interrelated Old World species in that group [10]. Our discovery of a species that is new to science, found feeding from the upper respiratory tract of humans in Perú, leads to a reanalysis of the phylogeny and classification of one clade of hirudinoid leeches, clarifying a single evolutionary origin of a group that specializes on mucous membranes and poses a threat to human health.

## Results

### Clinical Presentations

In 1997, a previously healthy six-year-old boy was admitted to a health center in Lamas province, department of San Martín, Perú complaining of frontal cephalgia. The patient's history revealed that, prior to admission, he frequently bathed in local lakes and natural streams. The patient reported neither bleeding nor respiratory distress. A 25 mm long leech was removed from the right nostril and preserved in formalin.

Again, in 1997, a 16-month-old boy was admitted to a local heath center in Yochegua province, San Francisco district, department of Ayacucho, Perú also complaining of frontal cephalgia and also without respiratory symptoms. It was ascertained that prior to admission the boy had bathed in small local lakes. A 60 mm leech was removed from the patient's nasal cavity, washed with saline solution and preserved in 10% formalin. Nasal bleeding continued for two days.

In 2007, a nine-year-old girl was admitted to La Merced hospital in Chanchamayo province, department of Junín, Perú following a two-week history of frontal cephalgia and a “sliding” sensation inside her nose. The patient's parents noticed a black worm moving inside her right nostril and sought medical attention. No other respiratory symptoms presented. The patient volunteered that she had been traveling in Satipo province, department of Junín, Perú where she frequently bathed in lakes, rivers and streams. Physical examination was remarkable only for nasal pain with hand pressure and a black mass inside the right nasal cavity. With some effort, a 65–70 mm black leech was removed without significant bleeding from the patient's nasal cavity, and was preserved in ethanol.

### Description

#### *Tyrannobdella* n. gen

One dorsal monostichodont jaw armed with few, large denticular teeth. Mouth velar with single slot for jaw. Ventrolateral jaws absent. Complete somite five-annulate. Cephalic eyespots, five pair in parabolic arc. Anus between last annulus and caudal sucker. Caudal sucker wider than posterior of body. Reproductive organs micromorphic. Feeds from mucosal surfaces of mammals. ZooBank LSID for the genus *Tyrannobdella* is urn:lsid:zoobank.org:act:43D55B49-C888-4D6B-AF6F-61238EC1339B. Type species: *Tyrannobdella rex* n. sp.

#### *Tyrannobdella rex* n. sp

Holotype: Preserved body length 44.5 mm, maximal width 0.95 mm, fixed and stored in 90% ethanol; dissected. Collected at La Merced Chanchamayo Junín, Perú in 2007 by Dr. Renzo Arauco-Brown; deposited in the Museum of Natural History of San Marcos University, Perú (catalogue number 2841).

Paratypes: Two mature specimens fixed in formalin and stored in 90% ethanol. Collected in departments San Martín and Ayacucho, Perú in 1997 by Dra. María Beltrán; one specimen deposited in the Enteroparasitology laboratory at the Peruvian Health Institute and another at the Museum of Natural History of San Marcos University, Perú (catalogue number 2842).

**Figure 2:**
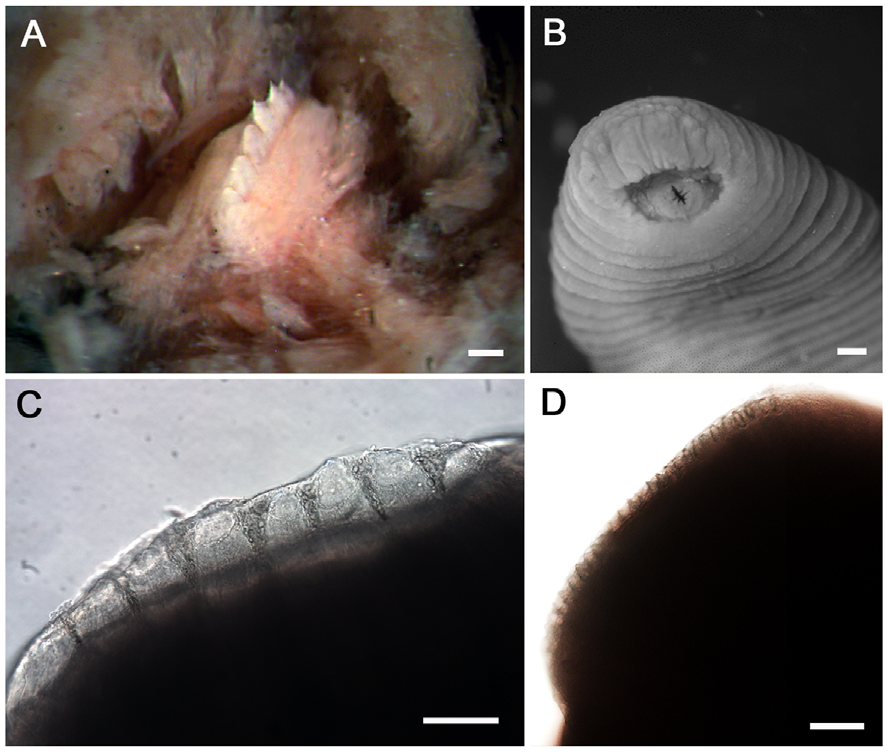
Comparative jaw morphology of *Tyrannobdella rex*. (A) Stereomicrograph of the single dorsal jaw of *T. rex* with large teeth. Scale bar is 100 µm. (B) *Tyrannobdella rex* anterior sucker exhibiting velar mouth and longitudinal slit through which the dorsal jaw protrudes when feeding. Scale bar is 1 mm. (C) Compound micrograph in lateral view of eight large teeth of *T. rex*. Scale bar is 100 µm. (D) Lateral view of jaw of *Limnatis paluda* illustrating typical size of hirudinoid teeth. Scale bar is 100 µm.

One dorsal jaw armed with eight large (up to 130 µm high) teeth forming a single (i.e., monostichodont) row (Fig. 2a,c). Two of eight teeth may be sub-cuticular and observable only with compound microscopy (Fig. 2c). Pharynx muscular and tubular. Crop from IX to XXV, first nine cecal pairs in IX through XIX, post-ceca extend blitaterally to XXV. First and second cecal chambers subdivided into two unequal sub-ceca with the larger being posterior, otherwise one cecal pair per somite. Intestine tubular, acecate.

**Figure 3:**
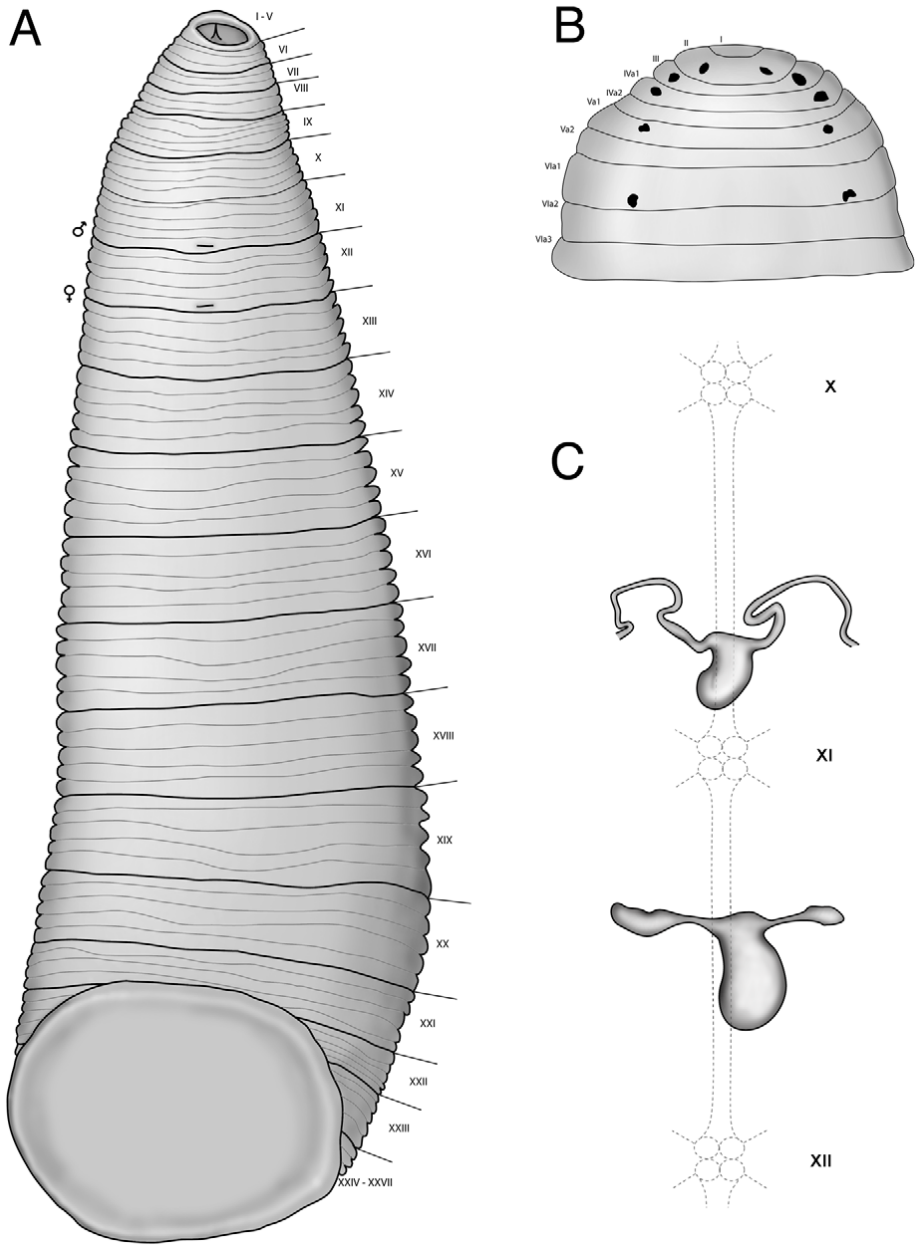
Comparative internal and external anatomy of *Tyrannobdella rex.* (A) Whole body ventral view illustrating annulation, relative size of the caudal sucker and relative position of gonopores. (B) Eyespot arrangement illustrated dorsally. (C) Male and female median reproductive anatomy.

Body muscular, uniformly pigmented brown to grey without stripes or other ornamentation after preservation. Papillae absent. Oral sucker small and velar (Fig. 2b). Oral opening central and dorsoventrally oval. Posterior sucker large, wider than posterior of body (Fig. 3a). Somites I - III uniannulate, somites IV – V biannulate, somites VI - VIII triannulate, somites IX - XXIV quinqueannulate, somites XXV triannulate with annulus a1 dorsally subdivided, somite XXVI biannulate with annulus a1 dorsally subdivided, and somite XXVII unianulate with a faint dorsal furrow visible. Anus between last annulus and caudal sucker. Eyespots, five pairs on II, III, IV a1, V a1 and VI a2, forming a parabolic arc (Fig. 3b). Male gonopore on XI b6, female gonopore on XII b6, gonopores separated by 1/2+4+1/2 annuli. Nephropores 17 pairs from VIII-XXV, each pair ventral on posterior margins of annulus b2 of somite.

Male and female reproductive organs extremely micromorphic, same size as or smaller than ventral ganglia (Fig. 3c). Penis sheath U-shaped, with initial posterior disposition and subsequent anterior procurrent portion leading to small epidydimis. Ejaculatory bulbs absent. Glandular prostate absent. Vagina present, U-shaped, no common oviduct and oviducts half the size of vagina. Vaginal cecum absent. Ovaries simple, bulbous.

The ZooBank LSID for the species *T. rex* is urn:lsid:zoobank.org:act:F8C0E97B-F525-4EB3-B11B-B8CBA1CB8F5F.

Etymology: *Tyrannobdella*: tyrannos (G.) – “tyrant” + bdella (G.) – “leech”; *rex*: rex (G.) – “king”.

Remarks: No other leech species is known to have but a single armed jaw with such large teeth. The reduced number of teeth, a caudal sucker wider than the posterior of the body, and preference for feeding on mucous membranes of mammals all indicate the placement of this new taxon within the family Praobdellidae among the genera *Praobdella, Myxobdella, Dinobdella, Limnatis,* and *Limnobdella*. *Pintobdella chiapasensis* (Caballero, 1957) similarly has few (six) teeth per jaw, albeit for each of three jaws. *Tyrannobdella rex* n.sp. unique in possessing only one jaw with eight large teeth (e.g., ∼five times the height of those in the genus *Limnatis*).

Members of the genus *Limnobdella* have two pairs of equal crop ceca in each gastric somite and an extended female reproductive structure. In comparison, *T. rex* has one pair of crop ceca per somite except in the first two chambers of the crop, which have two unequal crop ceca per somite. Overall, the relatively simple structure of the reproductive system in *T. rex* resembles that of *Limnobdella* species, but with considerable differences in size. *Tyrannobdella rex* is easily distinguished from members of the genus *Limnatis* by the possession of smooth jaws without salivary papillae, having a velar mouth without a longitudinal furrow in the upper lip, and by the simple minute reproductive structures. Also, species of *Limnatis*, like *Limnobdella*, have two equal pairs of crop ceca per somite, whereas *T. rex* has a single pair per mid-body somite.

Species of *Myxobdella* and *Praobdella* are morphologically similar to *T. rex* in possessing a velar mouth, a reduced number of teeth, and micromorphic reproductive structures. Unlike *Myxobdella* and *Praobdella*, each possessing two rows of teeth (i.e. distichodont) and three jaws, *T. rex* only possesses a single row (i.e. monostichodont) and one jaw. *Myxobdella* species are distributed throughout Southeast Asia and Africa, whereas *Praobdella* species are restricted to Africa. Besides differences in jaw armature, the genus *Myxobdella* is characterized by imperfect annulation and annulation furrows of unequal depth. In contrast, *T. rex* demonstrates 15 complete five-annulate mid-body somites with only the three most posterior somites having partially subdivided annuli. Species in the genus *Myxobdella* have gonopores separated by five or five and a half annuli, whereas *T. rex* has gonopores separated by 1/2+4+1/2 annuli. Species in the genus *Praobdella* lack the velar mouth and have at least seven annuli between gonopores.

### Phylogenetic analyses

**Figure 4:**
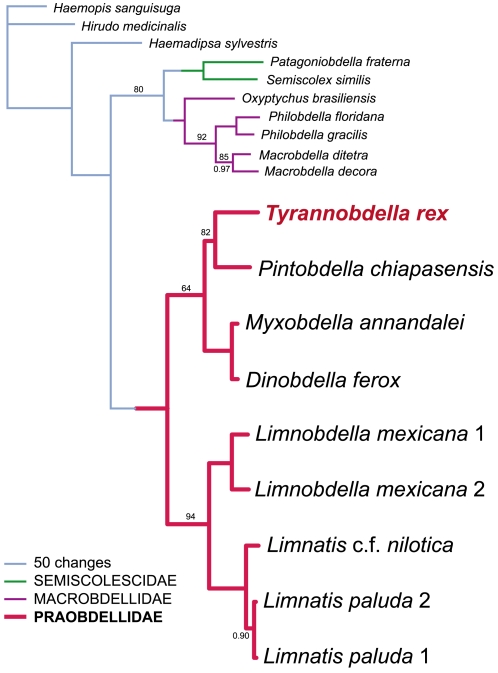
Single most parsimonious tree based on combined 18S rDNA, 28s rDNA, 12s rDNA, and COI datasets. The family Praobdellidae formed a well-supported monophyletic group of leeches that exhibits a predilection for mammalian mucosa. All groups received 100 percent bootstrap support and posterior probabilities of 1.00 except as noted on the tree. Branches are drawn proportional to amount of change.

The combined dataset included a total of 5256 molecular characters (18S rDNA: 2041 characters, 28S rDNA: 2189 characters, 12S mt rDNA: 367 characters, COI: 657 characters). Parsimony recovered a single tree with 2725 steps and the two runs of the Bayesian analysis had a harmonic mean of log likelihood values that averaged to–19830.23. Phylogenetic analyses recovered identical topologies regardless of method (Fig. 4).

A clade of hirudinoid leeches (including the genera *Limnobdella*, *Limnatis*, *Dinobdella*, *Myxobdella*, *Pintobdella* and *Tyrannobdella*), distinguished by their propensity for feeding on mammalian mucous membranes, was recovered as monophyletic with strong support (bs = 100; pp = 1.00). Sister to this was a strictly New World clade (bs = 80; pp = 1.00) comprising both the families Semiscolescidae (*Semiscolex* + *Patagoniobdella*) and Macrobdellidae (*Macrobdella* + *Philobdella* + *Oxyptychus*). The new Peruvian species, *T. rex*, was sister to the Mexican *P. chiapasensis* (bs = 82; pp = 1.00). *Dinobdella ferox* and *Myxobdella annandalei* Oka, 1917 were most closely related (bs = 100; pp = 1.00). Representatives of the Old World genus *Limnatis* formed a monophyletic clade sister to the Mexican genus *Limnobdella* (bs = 94; pp = 1.00). The clades (*T. rex + P. chiapasensis*) and (*D. ferox + M. annandalei*) also form their own clade sister to (*Limnatis* spp. *+ Limnobdella* spp.).

## Discussion

Hirudinoid leeches that show a preference for mammalian mucosal surfaces all appear to have descended from a common ancestor millions of years ago. Among these, the new species *Tyrannobdella rex* is the first from South America and one with a particularly unpleasant habit of infesting humans [12]. Another New World orifice-invading leech known from southern Mexico, *P. chiapasensis*, and sister taxon to *T. rex*, has only been found to parasitize the nasal passages of tapirs [13]. *Limnobdella* species from central and northern Mexico are known to be pests of livestock [14]. The consistency with which pain was reported by its victims may relate to the relatively enormous teeth *T. rex* has on its jaw.

Most of the documented cases of leech infestation are in tropical regions. Such cases are closely related to unsafe drinking water habits and people swimming in natural sources. It is in these situations that these worms enter the rectum, vagina or upper airway and attach to the mucosa [15]. A recent study revealed that the nose is the most common site of infestation (71%), followed by the hypopharynx (14%) [16]. Less often, leech infestations affect the lower airways causing haemoptysis, haematemesis, severe anaemia, airway obstruction or death [17]. While little is known of the symbiotic fauna for praobdellids, species of *Aeromonas* are known to inhabit the gastric ceca of various hirudinoid leeches [18], [19]. Insofar as praobdellids have been reported to remain attached for prolonged periods [1], there may also be a serious risk of bacterial infection to the extent that prophylactic antibiotic treatments is indicated in all cases of orificial hirudiniasis.

Several species of leech are known to invade human orifices, most notably various Old World species in the genera *Myxobdella, Praobdella,* and *Dinobdella*. Until now, the family Praobdellidae (*sensu* Sawyer, 1986) included only those three genera, two representatives of which were monophyletic in our analyses: *Myxobdella annandalei* and *Dinobdella ferox*. We found strong support for monophyly of that pair in a broader clade that also includes species of *Limnatis*, *Limnobdella*, *T. rex* and *P. chiapasensis*. This clade is defined not only by our molecular evidence, but also by three morphological and behavioral synapomorphies: reduced number of teeth–less than 12 in *Myxobdella, Dinobdella, Praobdella, Tyrannobdella*, and *Pintobdella*, and less than 40 in *Limnatis* and *Limnobdella*; the caudal sucker is wider than the posterior of the body; and a preference for feeding primarily on mammalian mucous membranes. The enlarged caudal sucker seen throughout this family may well be an adaptation that mediates attachment to moist mucous membranes [2]. Only once has a praobdellid been reported feeding opportunistically on amphibians when mammals were not available [20].

The systematics of the family Praobdellidae (*sensu* Sawyer, 1986) has been plagued by ill-defined groups and by substandard type specimens being the sole representatives for some species [1], [2]. The characteristics of the oral sucker, the color pattern, and the location of the gonopores seem to hold the most phylogenetic information among species, but organizing these species within genera has been confused. The Terrible Ferocious Leech, *Dinobdella ferox*, for example, was initially described as a species of *Whitmania*, a genus of non-bloodfeeding leeches more closely related to *Hirudo* [10]. Several morphological similarities have been noted [21] between *Praobdella radiata* Moore, 1958 and *Myxobdella africana* Moore, 1939, while *Praobdella guineensis* Blanchard, 1896, *Praobdella buettneri* Blanchard, 1896, and *Praobdella maculata* (Moore, 1939) have each been considered potential synonyms of *D. ferox* (Moore, 1958). It has generally been agreed that these taxonomic conundra will only be resolved with the addition of fresh specimens [2], [21]. Nonetheless, the monophyly in our phylogenetic analyses of the genera *Myxobdella, Dinobdella, Limnatis, Limnobdella, Tyrannobdella*, and *Pintobdella* agree with the morphological and behavioral synapomorphies observed throughout the clade suggesting that the family Praobdellidae should be expanded to include them all. In turn, this settles the problem faced by Phillips and Siddall [10], and allows Semiscolecidae Scriban and Autrum, 1934 to retain its traditional scope comprising non-bloodfeeding South American taxa and allows Macrobdellidae Richardson, 1969 to encompass the bloodfeeding genera *Macrobdella*, *Philobdella* and *Oxyptychus*.

Representatives of the genus *Praobdella*, preferably the type species *P. buettneri*, are sorely needed to definitively establish the relationships of members of the family Praobdellidae. *Praobdella buettneri* has not been collected since its description in 1896 (from Bismarksburg, Togoland, now the Togolese Republic) along with *P. guineensis,* which shares the same type locality [22]. Only external morphology was mentioned in Blanchard's (1896) description and the type specimens are long-since dried out making it difficult to relate them to newly collected material not found at the type locality. Additional species of this family that warrant scrutiny are *M. africana, Myxobdella sinanensis* Oka, 1925, *Myxobdella weberi* (Blanchard, 1897), *Myxobdella nepalica* Nesemann and Sharma, 2001, *P. maculata,* and *P. radiata*. Further collection efforts in Africa and Asia may yet successfully provide the required material, though our standard methods of attracting leeches to our exposed selves may prove awkward given their established propensity for particular anatomical feeding sites.

## Methods

Specimens of *T. rex* were collected from two states of Perú in 1997; one from a health center in Lamas province, department of San Martin, Perú, and one from a local heath center in Yochegua province, San Francisco district. Both of these specimens were preserved in formalin. A third specimen collected from a clinic in La Merced Chanchamayo Junin, Perú in 2007, was preserved in ethanol, and was the specimen chosen both for the holotype and for sequencing in these analyses. Specimens of *P. chiapasensis* were collected from forest streams leading to the lakes of Montebello, State of Chiapas, Mexico between 6 and 18 July, 2008. One *M. annandalei* was received in December, 2008 from Dharamsala, India. Tissue samples of *D. ferox* were collected on 13 April, 2008 in Taiwan. Examination of external and internal morphology was accomplished with a Nikon SMZ-U stereo microscope on whole and dissected specimens. Photographs were taken with a SPOT-RT digital camera. Drawings were made by superposition of vector art over images placed in Adobe Illustrator® 10 and Adobe Photoshop® 7.

### DNA sequencing and alignment

Tissue was collected from the caudal sucker in order to avoid contamination from host DNA in gastric or intestinal regions of the leech. DNeasy Tissue Kit (Qiagen Valencia, CA) was used for tissue lysis and DNA purification. Primers for the PCR amplification of nuclear 18S rDNA and 28S rDNA and mitochondrial cytochrome oxidase I (COI) and 12S rDNA gene fragments were adapted from published protocols [23], [24], [25], [26], [27] and are listed in Table 1. Amplification reactions of gene fragments were conducted using either Ready-To-Go PCR Beads (GE Healthcare, Piscataway, NJ) with 0.5 µl of each 10 µM primer, 1 µl DNA template, and 23 µl Rnase-free H2O (total volume 25 µl), or homemade Taq with 1.0 µl Taq, 2.5 µl MgCl, 2.5 µl 10x Buffer A, 1.0 µl dNTPs, 0.5 µl of each 10 µM primer, 2.0 µl template, and 15 µl H_2_O) (total volume 25 µl). PCR reactions were performed in an Eppendorf Mastercycler. The following amplification protocols were used: for 18S, 94°C (1 min) followed by 35 cycles of 94°C (30 sec), 49°C (30 sec), 68°C (2 min 30 sec) and final extension at 68°C (1 min); for 28S and 12S, 94°C (5 min), followed by 39 cycles of 95°C (1 min), 52°C (1 min), 70°C (1 min) and final extension of 72° (7 min); for COI, 94°C (1 min), followed by 30 cycles of 94°C (30 sec), 48°C (30 sec), 68°C (45 sec), 68°C (1 min) and final extension of 68°C (1 min). PCR amplification products were purified with AMPure™ (Agencourt Bioscience Corporation). Cycle sequencing reactions were performed with an Eppendorf Mastercycler® using 1 µl Big Dye™ Extender Buffer v3.1, 1 µl of 1 µM primer and 3 µl of cleaned PCR template (13 µl total volume). Sequences were purified by 70% isopropanol/70% ethanol precipitation and analyzed with an ABI PRISM® 3730 sequencer (Applied Biosystems). CodonCode Aligner (CodonCode Corporation) was used to edit and reconcile sequences. GenBank accession numbers are listed for sequences derived from each taxon in Table 2. Alignments of all genes were accomplished using the European Bioinformatics Institute server for MUSCLE v. 3.7 applying default settings [28].

**Table 1:** Genes and primer sequences used in phylogenetic analyses.

Gene	Primer Name	Primer Sequence	Reference
*Nuclear*			
18S rDNA			
1	A	5′-AACCTGGTTGATCCTGCCAGT-3′	Apakupakul et. al., 1999
	L	5′-CCAACTACGAGCTTTTTAACTG-3′	Apakupakul et. al., 1999
2	C	5′-CGGTAATTCCAGCTCCAATAG-3′	Apakupakul et. al., 1999
	Y	5′-CAGACAAATCGCTCCACCAAC-3′	Apakupakul et. al., 1999
3	O	5′-AAGGGCACCACCAGGAGTGGAG-3′	Apakupakul et. al., 1999
	B	5′-TGATCCTTCCGCAGGTTCACCT-3′	Apakupakul et. al., 1999
28S rDNA			
1	28srD1a	5′-CCCSCGTAAYTTAAGCATAT-3′	Prendini et al., 2005
	28sB	5′-TCGGAAGGAACCAGCTAC-3′	Whiting, 2002
2	28sA	5′-GACCCGTCTTGAAGCACG-3′	Whiting, 2002
	28SBout	5′-CCCACAGCGCCAGTTCTGCTTACC-3′	Prendini et al., 2005
3	28srD5a	5′-GGYGTTGGTTGCTTAAGACAG-3′	Whiting, 2002
	28srD7b1	5′-GACTTCCCTTACCTACAT-3′	Whiting, 2002
*Mitochondrial*			
12s rDNA			
	12Sa	5′-AAACTAGGATTAGATACCCTATTAT-3′	Simon et al., 1994
	12Sb	5′-AAGAGCGACGGGCGATGTGT-3′	Simon et al., 1994
COI			
	LCO1490	5′-GGTCAACAAATCATAAAGATATTGG-3′	Folmer et al., 1994
	HCO2198	5′-TAAACTTCAGGGTGACCAAAAAATCA-3′	Folmer et al., 1994

**Table 2:** Taxa used for the phylogenetic analyses, collection localities, and GenBank accession numbers.

Taxon	Locality	GenBank Accession Numbers			
		18S	28S	12S	CO1
Ingroup					
*Dinobdella ferox*	Taiwan	GU394006	GU394010	GU394002	________
*Limnatis* cf. *nilotica*	Namibia	GQ368795	GQ368774	GQ368815	GQ368754
*Limnatis paluda* 1	Afghanistan	GQ368796	GQ368775	________	GQ368755
*Limnatis paluda* 2	Israel	AY425470	AY425389	AY425430	AY425452
*Limnobdella mexicana* 1*	Mexico	GQ368798	GQ368777	GQ368816	GQ368756
*Limnobdella mexicana* 2*	Mexico	GQ368799	GQ368778	GQ368817	GQ368757
*Myxobdella annandalei*	India	GU394007	GU394011	GU394003	GU39414
*Pintobdella chiapasensis*	Chiapas, Mexico	GU394008	GU394012	GU394004	GU394015
*Tyrannobdella rex*	Perú	GU394009	GU394013	GU394005	GU394016
Outgroup					
*Haemadipsa sylvestris*	Vietnam	AF116005	AY425373	AY425416	AF003266
Haemopis sanguisuga*	Sweden	AF099941	AY425381	AF099960	AF462021
Hirudo medicinalis*	BioPharm, UK	AF116011	AY425385	AF099961	AF003272
Macobdella decora*	MI, USA	AF116007	AY425390	AY425431	AF003271
*Macrobdella ditetra*	GA, USA	AY425471	AY425391	AY425432	AY425453
*Oxyptychus brasiliensis*	Brazil	AY425473	AY425398	AY425436	AY425455
*Patagoniobdella fraterna*	Chile	AY425477	AY425405	AY425441	AY425459
Philobdella floridana*	SC, USA	DQ097210-13	DQ097201-14	DQ097226	DQ097219-22
*Philobdella gracilis*	LA, USA	DQ097209	DQ097200	DQ097225	DQ097218
*Semiscolex similis*	Bolivia	AY425475	AY425402	AY42543	AY425475

Type species of genera are indicated with an asterisk.

### Phylogenetic analyses

A total of 17 species comprising 19 terminals were used in the analyses with *Hirudo medicinalis* specified as the outgroup (Table 1). Phylogenetic analyses were conducted using two approaches: Parsimony and Bayesian Inference (BI). Parsimony analyses were conducted in TNT v 1.1 [29] using 10 replicates of random taxon addition, sectorial searching, the Ratchet [30], and tree-bisection-reconnection branch swapping for each gene as well as for the combined dataset (18S, 28S, 12S, COI). Bootstrap values for combined analyses were obtained using 10 heuristic pseudoreplicates and the same analytical settings. Bayesian analyses were conducted in MrBayes v. 3.1.2 [31]. The data were partitioned by gene for 18S, 28S, 12S, and by codon position for COI (three partition; 3p). A GTR+I+Γ model was applied to each unlinked data partition based on the Akaike Information Criterion [via ModelTest v. 3.7; 32, 33]. For the Metropolis-Coupled Markov Chain Monte Carlo (MCMCMC) analyses, default prior distributions of parameters were used twice with one cold chain and three hot chains for 10 million generations and sampled every 1000^th^ generation. The BI analyses burned-in before 100,000 generations. Split frequencies of the standard deviation of simultaneous BI analyses were well below 0.01. As such, the burn-in was set to discard the first 100,000 generations, leaving 9,900 trees sampled for estimation of posterior probabilities.

### Nomenclatural Acts

The electronic version of this document does not represent a published work according to the International Code of Zoological Nomenclature (ICZN), and hence the nomenclatural acts contained in the electronic version are not available under that Code from the electronic edition. Therefore, a separate edition of this document was produced by a method that assures numerous identical and durable copies, and those copies were simultaneously obtainable (from the publication date noted on the first page of this article) for the purpose of providing a public and permanent scientific record, in accordance with Article 8.1 of the Code. The separate print-only edition is available on request from PLoS by sending a request to PLoS ONE, 185 Berry Street, Suite 3100, San Francisco, CA 94107, USA along with a check for $10 (to cover printing and postage) payable to “Public Library of Science”.

In addition, this published work and the nomenclatural acts it contains have been registered in ZooBank, the proposed online registration system for the ICZN. The ZooBank LSIDs (Life Science Identifiers) can be resolved, and the associated information viewed, through any standard web browser by appending the LSID to the prefix “http://zoobank.org/”. The ZooBank LSID for this publication is: urn:lsid:zoobank.org:pub:8D431ED1-B837-4781-A591-D3886285283A
